# Constructing the Elements of the “Recovery in” Model through Positive Assessments during Mental Health Home Visits

**DOI:** 10.3390/ijerph15071441

**Published:** 2018-07-09

**Authors:** Suvi Raitakari, Suvi Holmberg, Kirsi Juhila, Jenni-Mari Räsänen

**Affiliations:** Faculty of Social Sciences, University of Tampere, Tampere 33014, Finland; suvi.holmberg@uta.fi (S.H.); kirsi.juhila@uta.fi (K.J.); jenni-mari.rasanen@uta.fi (J.-M.R.)

**Keywords:** recovery, supportive communication, positive assessments, interaction, mental health, home visit

## Abstract

The study provides a categorization of the different elements of the “recovery in” model (RIM). The objective is to analyze elements of RIM in positive assessments during home visit interactions. RIM approaches mental illness as a long-term condition that people live with in their daily lives in their communities. The model emphasizes the rights of all citizens to be full members of their communities regardless of their mental health problems or other difficulties. Positive assessments are professionals’ encouraging evaluations of the activities, situations, or inner conditions expressed by the clients. They are essential in creating supportive professional-client communication. The data analyzed in this study consists of 17 audio-recorded home visits of 10 different clients. Home visits were provided by a mental health floating support service in 2012. The data was analyzed using coding and ethnomethodological interaction research (EIR). As a result RIM is divided into two upper-categories: “Encouraging Doing the Right Thing” and “Encouraging the Right Kind of Personal Growth”. These categories include a wide spectrum of elements that are relevant for the client’s agency in the community. The elements embed the client’s performance in everyday routines and the client’s progress in becoming a skillful, knowledgeable, and involved agent in the community. The categorization of the elements of RIM could be used in educating practitioners to identify and operationalize RIM in mental health home visits.

## 1. Background

### 1.1. “Recovery in” Model and Home as a Scene of Service Delivery

The recovery movement has developed from a number of medical and social sources. A common aim of the movement is to promote high quality and user-centered mental health services. The movement promotes well-being, self-determination, and full citizenship for individuals suffering from mental health problems [1,2,3]. Its origins can be located in the addiction treatment self-help movement [4]. The recovery movement also has origins in the independent living and the Civil Rights Movements of the 1960–1970s and to the later mental health consumer movement. The recovery movement has had a central influence on current mental health policy and practice in many Western countries (e.g., [4,5,6]). It has been more influential in English-speaking countries than in continental Europe. However, the prevalence of the movement has expanded in Europe in recent years (e.g., [7,8]).

The recovery movement is supported by evidence-based research results and positive personal testimonies. These show that a great majority of people can recover from mental health difficulties completely or partly. Recovery allows people to live a normal life in the community, at least with professional and informal support [2,6,9,10,11]. Recovery comprises of external, internal, and psychosocial factors [3,12]. Jacobson and Greenley [9] describe the multi-layered concept of recovery:

Recovery is variously described as something that individuals experience, that services promote, and that systems facilitate, yet the specifics of exactly what is to be experienced, promoted, or facilitated—and how—are often not well understood either by the consumers who are expected to recover or by the professionals and policy makers who are expected to help them.

The research literature provides various classifications of the elements of recovery based on focus groups, first person accounts, and literature reviews (e.g., [13,14]). Recovery has been divided into the following elements: reconnecting with oneself, others, and time [15]. It has been related to the reawakening of hope, acceptance, active participation, and coping as well as to reclaiming a positive sense of self and meaningfulness in life. Recovery has also been defined as a complex and nonlinear journey that involves support and partnership [16].

In addition, the literature on this topic has presented distinctions between the clinical and rehabilitation model of recovery [12,17]. The clinical model approaches mental health issues from a medical point of view. This model’s central aim is to cure the client and enable him/her to disengage from mental health services. It describes elements of symptom remission and symptom-free conditions. In contrast, the rehabilitation model approaches recovery as a subjective survival process. This is often understood as a long journey toward better well-being and participation. In the rehabilitation model of recovery the aim is to improve one’s everyday life despite the limitations imposed by enduring mental health difficulties and social disadvantages [4,17].

Based on the clinical and rehabilitation models of recovery, Davidson and Roe [10] distinguish between the “recovery from” and the “recovery in” mental illness recovery models (see also [2]). The “recovery from” model emphasizes becoming cured from mental illness and restoring full everyday functioning. The “recovery in” model (RIM) instead approaches mental illness as a long-term condition that people live with in their daily lives in their communities. The viewpoint of RIM is less diagnostic and more social and existential in nature (see also [14,18,19]). RIM emphasizes the right of all citizens to be full members of the community regardless of their mental health problems or other difficulties. This right is realized when people live in ordinary environments and neighborhoods.

RIM emphasizes that a permanent home is a basic human right for everyone. The delivery of at-home health and social services has increased along with deinstitutionalization over recent decades (e.g., [20,21]). Accordingly, clients’ private environments have become locations of service provision and client-professional service interactions [22,23].

Although RIM is introduced as a policy and a framework for mental health practice, there is little consensus about what RIM actually means and how it is practiced in community mental health [4,10,24]. Therefore, it is important to conduct more research on the elements of RIM. It is essential to explore the gaps in knowledge on how the elements are constructed in professional-client interaction in mental health home visits.

### 1.2. Positive Assessments and Supportive Communication

Researchers have defined positive assessments in various ways (e.g., [25]). For the purposes of this study the definition introduced by Janet Holmes will be used: “a speech act which explicitly or implicitly attributes credit to someone other than the speaker, usually the person addressed, for some ‘good’ (possession, characteristic, skill, etc.) which is positively valued by the speaker” [26] (p. 485). Positive assessments presented in client-professional interaction provide optimistic and encouraging evaluations of the activities, situations, or inner conditions expressed by the clients. These evaluations can include longer utterances like, “You have made a great job in cleaning the floor” or shorter responses such as ”great”, ”good”, ”wonderful”, ”nice”, ”important” [27] (pp. 122–123), [28] (p. 796).

Positive assessments form a substantial part of supportive communication. Supportive communication is especially meaningful when a person is in distress and struggles with difficulties in life [27]. It may be expected that people struggling with mental health issues encounter various challenges that require social and practical support (see [29]). Positive assessments are commonly intended to express empathy, understanding, acceptance, and encouragement in order to restore a person’s strength and self-esteem [27] (p. 119), [28] (p. 795), [30]. The positive assessments often display and aim at alignment with a person it is addressed to. Positive assessments are a valuable professional skill in social and mental health care.

Several scholars (e.g., [31,32,33]) have examined in detail how assessments can be accomplished through human conversation and interaction. More recently, positive assessments have been studied in various institutional support work contexts, such as in the context of home birth help line interactions [25], in other medically related encounters [27], and in mental health consumer-run help lines [28]. Research has described (a) how positive assessments are designed, (b) how they are responded to in interaction, and (c) what they are used for in the institutional support work.

Jones [27] (p. 122) writes how “one way to be verbally supportive is to react positively to talk involving information that can be interpreted as ‘good news’” [27] (p. 122). Practitioners routinely re-orient to given information depending on whether it is good or bad news. When a recipient of information interprets the information as “good news”, she/he offers a positive assessment to the person delivering the information. In this study positive assessments are approached as professionals’ responses to clients’ expressions that the professionals interpret as “good news”.

Positive assessments are value-laden and consist of cultural expectations of a “good” citizen and “normal life” [27]. Positive assessments are always constructed and put to use according to cultural norms and normality. This is presumably the case when the elements of RIM are constructed in mental health home visit interaction with positive assessments.

There is intensive research literature on supportive communication and positive assessments. Similarly, the concept of recovery is widely discussed and applied in (mental) health and social care research. In this study, these two research branches are combined. This enables a deeper understanding of RIM as a supportive and value-laden practice that is put to practice in home visit interaction.

## 2. Research Design

### 2.1. Research Question and Aim

The study is based on the following questions: What kinds of elements of RIM exist in positive assessment sequences? How are these elements accomplished in home visit interaction? This study is motivated by the fact that although RIM has become a widely adopted practice among mental health professionals and clients, there is no consensus on what it actually entails and requires (e.g., [9,10,13]). The aim is to provide a categorization of the different elements of RIM and to demonstrate how these elements are accomplished in home visit interactions. To our knowledge, there is scarce research (e.g., [29]) that explores how RIM as a framework is presented and used in professional-client interactions.

### 2.2. Setting and Data

The research setting for this study is a floating support service run by a local, non-profit mental health organization that has developed community care in Finland since the 1970s. The organization’s principles are in line with RIM, which creates the framework for the organization’s mental health work. Floating support refers to services provided in clients’ homes. Home visits can also include activities in the community, such as going for a walk or to a cafeteria.

The service is targeted at people aged 18–65 and its practice is based on values of voluntariness, client-centeredness, and psycho-educative approaches. The service is generally fixed-term but provides opportunity for fairly long-term, supportive professional-client relationships. The professionals in this study are qualified mental health or social care workers with broad experiences in mental health, substance abuse, and family work. The clients struggle with a variety of mental health difficulties such as psychotic disorders, anxieties, depression, neurotic behavior, isolation, and social fears. Their medical psychiatric treatment is commonly managed by local outpatient psychiatric clinic. The aim of the floating support service is to enable, support, and ensure that clients can maintain their everyday lives at home and in their communities. During the course of a home visit, the following topics are usually discussed: psychosocial conditions, medical care, life management, self-esteem, sense of security, financial issues, social network, meaningful activities, and attendance in group activities and substance abuse treatment.

The professionals first invited their clients to take part in the study. If the clients agreed, the researcher contacted them and asked for permission to audio-record their home visit(s). All clients of the floating support service who were willing and able to participate were successfully recruited in the study. This resulted in a total of 10 participants (6 men and 4 women) from about 50 clients of the floating support service. As a small thank you for participation, the clients were given afterwards a 10-euro gift card to a hamburger restaurant.

The data consists of 17 audio-recorded home visits conducted by the floating support service in 2012. It includes two visits to five participants’ homes and one visit to four participants’ homes, and one participant was visited three times. In the data all of the mental health professionals who conducted the home visits were women. Because the focus of the study was on home visit interaction “in situ”, ages or diagnoses were not systematically collected. All clients participating in the study were adults and native Finnish citizens suffering from severe but not acute mental health problems. Since they were all clients of the floating support service, they were at a similar phase in their recovery processes. The clients were in good enough condition to live independently in the community, although they needed some professional support with everyday living.

The home visits lasted from 21 min to 47 min (in total 627 min). The transcribed audio-recorded data consists of 174 pages of text (Verdana font, size 8, line spacing single). The researcher audio-recorded 11 of the home visits and the professionals recorded six of the visits. There may be concerns that the presence of the researcher had an influence on home visit encounters. However, the transcribed data suggests that it is difficult to determine differences between the home visits with the researcher present and with the researcher absent. The data is to be considered as “naturally occurring” as possible in the given circumstances.

### 2.3. Research Ethics

When studying “naturally occurring” mental health service encounters in community settings, ethical principles such as anonymity, self-determination, voluntariness, and avoidance of harm to participants require special consideration. The research followed the guidelines of the National Advisory Board on Research Integrity, which defines ethical principles of research in the humanities and social and behavioral sciences [34]. The study was reviewed in November 2011 by The Ethics Committee of the Tampere Region that provides ethical reviews on non-medical research in the field of the human sciences; the Committee did not find any ethical problems in the study. Both professionals and clients were informed about the study and both oral and written consents were obtained. The professionals and the researcher together ensured that each client was in such a condition that it was safe for him/her to take part in the study. All the data presented in this article are carefully anonymized and treated with respect for the participants and their viewpoints. The whole data corpus is stored in a locked cabinet and on a password-protected computer.

### 2.4. Method and Analysis

Coding was used (e.g., [35]) to differentiate RIM related categories that were identified from home visit data. Then ethnomethodological interaction research methods (EIR) (e.g., [36,37,38,39]) were used to analyze the positive assessment sequences in detail. EIR studies the sequences of the conversations, the orientations of the interlocutors, and the collaborative meaning making in the interactions (in this study related to RIM) (e.g., [40,41,42]).

In the *coding phase* all the professionals’ positive assessments were identified from the transcribed data by utilizing the qualitative data analysis software Atlas.ti (see also [35]). In total 96 positive assessment sequences were coded from the data. These assessments comprise 27 out of the 174 pages (15, 5%) of the transcribed data corpus. The coding of transcribed data was discussed and checked jointly by the authors until there was consensus regarding the identification of the elements of RIM in home visit interactions.

In *the interaction analysis phase*, EIR was utilized to analyze the positive assessment sequences in detail. In this way it was possible to grasp how the sequences were structured and how the elements of RIM were accomplished by the clients and professionals.

## 3. Results

### 3.1. Elements of RIM Embedded in Positive Assessments

The home visit data contains both minimal (e.g., “very good”) and extended positive assessments (e.g., “you have been behaving very wisely”). These positive assessments were embedded in sequences in two ways: (a) as responses to client-initiated “good news” statements or (b) as responses to client-provided “good news” statements that follow the professionals’ question or advice. The coding of sequences resulted in two upper- and eight sub-categories that contain the elements of RIM (see Table 1). This section describes the upper- and sub-categories of RIM constructed in the positive assessments provided during home visits.

Positive assessments are primarily used to encourage the client to conduct everyday routines and to behave in such a manner that resonates with the ideals of RIM. Accordingly, the first upper-category was titled “Encouraging Doing the Right Things”. This upper-category covers 65 of the positive assessment sequences. It contains the following sub-categories: “taking care of everyday matters”, “taking care of home”, “taking care of oneself”, and “behaving in a normal way in the community” (see Table 1).

Some of the positive assessment sequences were provided to support the clients’ personal independency, goals, and plans in life (beyond just managing everyday routines and norms). This resulted in the development of another upper-category, “Encouraging Right Kind of Personal Growth”. This category contains 31 positive assessment sequences. It includes the following sub-categories: “doing life planning”, “doing illness-management”, “being self-governing and knowing agent”, and “being skillful community member”.

Some sub-categories are similar to each other thematically, but they represent different aspects of client agency promoted by RIM. In the first upper-category, the sub-categories are related to assessments that encourage clients to carry out routine tasks of normal everyday life. Sub-categories under the second upper-category focus more on more profound aims of a self-governed person and active citizenship. Table 1 summarizes the upper and sub-categories of RIM defined in this study.

### 3.2. Elements of RIM in Home Visit Interaction

In the following sub-sections, one example of each positive assessment sequence sub-category is analyzed in detailed in relation to the research questions. The EIR-based analysis demonstrates how the elements of RIM are constructed and used in home visit interaction. The analysis also reveals cultural expectations about what the right kind of agency means for a person to be “in recovery”. Before each data example, some background knowledge about the client’s situation and the functions of the home visit are provided.

#### 3.2.1. Encouraging Doing the ‘Right’ Things

• Taking Care of Everyday Matters

In this data example a professional and a female client are discussing the client’s financial issues. The client has a difficult habit of making impulsive mail orders, which have a negative influence on her ability to take care of regular monthly payments.

1. PROFESSIONAL: So, when you get the unpaid (rent) payments taken care of, you may then try to put a little money aside.
2. CLIENT: And in July I will already pay July’s rent, so then it will be leveled!
3. PROFESSIONAL: Oh right, wonderful!
4. CLIENT: Yes, I counted it already in advance. This month I will still pay March’s (rent), so it is already paid.

The positive assessment sequence starts with the professional’s advice and suggestion that the client should put some money aside after the rent payments have been addressed (Turn 1). The client responds by discussing her current payment plan (Turn 2). The professional hears this as “good news” and responds with the minimal positive assessment “*Oh right, wonderful*” The client marks the positive feedback by providing more information about her payment schedule. What makes the client’s accounts (Turns 2 and 4) “good news” from a RIM-informed perspective? The client’s accounts indicate that her housing is now secured and sustained. She had experienced earlier challenges with taking care of money matters, as the professional mentions when she refers to “unpaid rent payments” (Turn 1), and the client considers it important to tell to the professional about her payment plan in the subsequent interaction. Additionally, the client portrays herself as a planning, systematic, and responsible agent, which can be seen as positive characteristics for a person “in recovery” process. The client is doing things that “ordinary” person would do, i.e., she pays her rent and shows a capacity to plan her actions. Both are important prerequisites for living independently and are thus considered “good news” for the professional. Supporting clients in their efforts to take care of their everyday matters and making positive assessments concerning these activities are crucial in RIM-oriented mental health work.

• Taking Care of the Home

The main focus during the home visit in this example is the female client’s health issues, including frequent urinary tract infections and weight loss. Additionally, the visit included discussion about the frequency of ordinary everyday chores.

1. PROFESSIONAL: How about the dishes?
2. CLIENT: The dishes have been taken care of.
3. PROFESSIONAL: The dishes have been taken care of. Okay, good, good.

This example represents a positive assessment sequence that starts when the professional asks a question. For the professional the “good news” is that the client states that the dishes “*have been taken care of*”. The professional constructs a repetitive, minimal positive assessment: “*The dishes have been taken care of. Okay, good, good*” (Turn 3). The positive assessment can be interpreted as a form of encouragement for the client to continue the activity that is seen as ‘the right’ thing to do to take care of the tidiness of the home. The professional’s opening question (Turn 1) indicates that the client has had problems doing the dishes in the past and that it is something to be checked on during the home visit. Because the floating support service is targeted at persons living in their own housing, in normal flats, taking care of home is an essential element of the “recovery in” process and something that is assessed by the professionals.

• Taking Care of Oneself

In the next example, the professional is visiting a female client. During routine discussions concerning the client’s daily activities, weekly schedule, and mental and physical condition, the client begins to brush her teeth.

1. PROFESSIONAL: All right, good. This would also be a kind of an important thing to remember to take care of brushing your teeth every day.
2. CLIENT: Yes, I try.
3. PROFESSIONAL: Keep the plug in the wall socket so that the toothbrush will recharge.
4. CLIENT: Yes, I always try.
5. PROFESSIONAL: All right, that’s already a lot.
6. CLIENT: Yes.

In the beginning of the positive assessment sequence, the professional advises and reminds the client about the importance of brushing her teeth daily. The client responds by accepting the given advice and expresses that she will try to act accordingly (Turn 2). The professional continues by giving a second round of advice regarding how to operate an electronic toothbrush (Turn 3), and as a response the client again emphasizes that she is trying to remember to keep the electric toothbrush plugged in to charge (Turn 4). The professional treats the client’s response as “good news” and something worth strengthening via extended a positive assessment: “*All right, that’s already a lot*” (Turn 5). The client agrees with this evaluation by saying “yes”. It can be assumed that successful mental health work based on RIM relies on “trying” clients who are ready to make an effort to improve their well-being and coping. “Trying” can be interpreted as a culturally valued characteristic. It indicates that the person wants to do things that are widely culturally accepted as “right”, yet s/he is not always capable to carry them out. During the home visits, the floating support service professionals support and direct the clients in many ways regarding personal hygiene, clothing, nutrition, eating habits, and exercise. Thus, taking care of oneself is a central element in the “recovery in” process.

• Behaving in A Normal Way in the Community

In the next example, a female client and a professional are discussing the client’s impulsive and manic behavior, and its effects on the surrounding community.

1. PROFESSIONAL: In what kind of condition have you been, all things considered?
2. CLIENT: Pretty good.
3. PROFESSIONAL: As there have been changes in medication and in other things?
4. CLIENT: Yes, I haven’t been talking any rude things and I have not been ringing anyone’s doorbell.
5. PROFESSIONAL: So, you have been behaving very wisely, yeah?
6. CLIENT: I still sang sometimes on the balcony. I apologize if someone hears.
7. PROFESSIONAL: Yes, it easily resounds, if you sing loudly because here is so much those high-rise apartment buildings.

The positive assessment sequence starts with a question from the professional addressing the client’s current condition. The client disclosures that she has been feeling ”*pretty good*” (Turn 2) and how this can be observed from changes in her behavior (Turn 4). The professional interprets the client’s description of her current state as “good news” and responds with an extended positive assessment: “*So, you have been behaving very wisely, yeah*”. Interestingly, the client responds by “confessing” that the positive behavior change has not been total and she apologizes for her possible disturbing behavior (Turn 6) as a ”good” and ”normal” community member should do. One crucial element of RIM is to support the clients to act in relation to other community members and close ones as is seen acceptable and normal. In this way, RIM-based orientation aims to ease the client’s integration to the community.

#### 3.2.2. Encouraging Right Kind of Personal Growth

• Doing Life Planning

In the next example, a male client and a professional are discussing plans for the approaching summer. The client currently studies at a Bible academy for four hours per day. He is wondering whether or not it would be too demanding for him to simultaneously continue his studies and to have a summer job.

1. PROFESSIONAL: Yes, so there would probably be a possibility, let’s say to be a camp leader.
2. CLIENT: Mm, and then also the Lutheran church has these summer camps where they always need staff for the summer. And then there was, the city also had something (a work offer), I can’t now remember what it was, but something I should know how to do.
3. PROFESSIONAL: Yes. But you already have experience as you have been going to for example a center or what was there where you were arranging those group activities and everything like that, so yes, for sure you have competences. But then of course according to your strengths it is important that you do not take too large a task for yourself.
4. CLIENT: Yeah, yes that’s it. But as all is just fine now, that I have four hours a week there and additionally all kinds of things going on, so in the end it is almost like having a part-time job.
5. PROFESSIONAL: Indeed.

The positive assessment sequence starts with professional advice that addresses the client’s future work options (Turn 1). The client continues discussing and planning his future life plans that could involve working for the Lutheran Church (Turn 2). The professional’s extended positive assessments (Turn 3) of “*you have already experience*”, “*so yes, for sure you have competences*” encourages the client to see himself as a competent and fit person for the planned summer activities. In that way the professional strengthens the client’s agency and image of being a capable person. However, in the end of Turn 3 the professional expresses caution to avoid overextending beyond the client’s strengths. The client responds to this reservation by saying that he is a strong agent capable of at least holding a part-time job (Turn 4) to which the professional responds with a minimal positive assessment “*Indeed*” that expresses acceptance and agreement. The example demonstrates one essential element of RIM: strengthening the client’s self-governing agency by constructing positive visions of the future tasks and responsibilities. The professional expresses that the client is capable of carrying out his plans, but at the same time notices and expresses the possible risks of exhaustion and failure. The image of a strong, active agent that plans his/her future also resonates with the Western idea of an active citizen.

• Doing Illness Management

In the next example, a professional is visiting a male client who has heavily misused pain and sedative drugs in his previous history. During the home visit, the client is telling a story about how he managed to resist a desire to go to the bar.

1. CLIENT: Well, one example, once I thought I was coming from (a name of a residential area). I had had very much all sorts of things going on, and so... Then I got this terrible... nearby is a small bar and it ‘pulled’ me like a magnet and I was thinking what will I do now... that things will only go badly, so I called my dad and right away when I told the situation to him, it ‘snapped’, it stopped like a ‘cold turkey’ that ‘pulling’. And after that I got to come home peacefully. That is effective.
2. PROFESSIONAL: Indeed, that is your way to manage, and so yes, it really does the trick.

The client describes a difficult situation and how he managed successfully to overcome it by calling his dad (Turn 1). For the professional the story is “good news” as she gives the client encouraging feedback by providing an extended positive assessment: “*Indeed, that is your way to manage, and so yes, it really does the trick*”. By doing this, the professional emphasizes that the client possesses successful techniques to manage his addictions, and thus is capable of governing himself in a favorable way and becoming a strong agent in the struggle against obsessions. This reflects an important element of RIM, which is to strengthen clients’ agency by communicating support for their ability to manage illnesses and addictions.

• Being a Self-Governing and Knowing Agent

In the next example, a professional and a male client are discussing a previously planned excursion to a day-activity center. The professional has identified that the client needs more social activities included in his weekly schedule to decrease social isolation. During the conversation, the professional begins to wonder how eager the client truly is to take part in the considered activity.

1. PROFESSIONAL: Right. So, just tell straight out, are you going to go and become familiar with the activity center just to please us support workers, or are you going there because you want to?
2. CLIENT: Perhaps mainly to please the support workers.
3. PROFESSIONAL: Okay. So, what is yours, honestly, your will, your desire and your wish?
4. CLIENT: That, at the moment I don’t want to go (to the day activity center).
5. PROFESSIONAL: You don’t?
6. CLIENT: I would not want to go.
7. PROFESSIONAL: Okay, it is good that you said this at this point, there is no point that we go to the activity center, that you go there, if you go there just to please me, or you assume that it is something that I would wish you to do. So, there is no use, it doesn’t lead anywhere.
8. CLIENT I don’t know about the end of the summer, if then I would be more willing.
9. PROFESSIONAL: That is really good, very good that you said it, what do you think and feel about that, what feelings do you have.

The example starts with the professional asking a question to determine the client’s real will, desires, and wishes concerning the trip to the activity center (Turns 1 and 3). In the interaction, the professional is “fishing” for the client’s real opinion three times (Turns 1, 3 and 5), and accordingly the client expresses three times (Turns 2, 4 and 6) that his motive is just to please the professionals and that he actually does not want to go along with the current plan. For the professional a “resisting client “who expresses his true thoughts is “good news”. Thus, she responds by providing extended positive assessments: “*Okay, it is good that you said it at this point*”; “*That is really good, very good that you said it, what do you think and feel about that, what feelings do you have.*” It can be interpreted from the interaction that for the “recovery in” process, a client who is too adapting and pliable is a problem, because one aim of the RIM is to restore a client’s self-determination and right to make decisions according to his/her own will. This element of RIM is accomplished in this professional-client interaction first by prompting the client to form an independent view and then by justifying his (resisting) opinion by responding to it with a positive assessment.

• Being Skillful Community Member

In the next example, the female client is organizing her jewelry-making equipment when the professional begins to discuss this special interest and skill that the client has. One main theme of the home visit is the difficulty that the client faces in keeping her home in order and how this increases her stress level.

1. PROFESSIONAL: How does it feel being asked to take part in that kind of activity (to be one of the leaders of a bauble course)?
2. CLIENT: Yes, I will go alright, or there will probably be more than one (course), because probably not everyone will necessarily fit in.
3. PROFESSIONAL: But, so, after all, you can be proud of yourself that you have been asked to that kind of, that in my opinion, it is a great thing. Surely it will be fun.
4. CLIENT: Yes, at least something a bit different.
5. PROFESSIONAL: Yeah. And as you apparently are good at this jewelry making, after all, it is great, then you have a possibility to share your own expertise with others.
6. CLIENT: This organizing thing at home comes at a good time, now all the missing pieces of jewelry may be found.

Once again a positive assessment sequence begins with the professional’s question, this time concerning the client’s feelings and inner thoughts about the request to be a leader of a leisure time course (Turn 1). The client responds with a neutral statement regarding the opportunity (Turn 2) to which the professional responds with an extended positive assessment that is meant to strengthen the client’s self-esteem and to emphasize the significance of the “good news” (i.e., the client has been asked to take a responsible role): “*you may be proud of yourself that you have been asked to do that kind of, that in my opinion, it is a great thing. Surely it will be fun.*” The client does not directly reflect the given positive feedback but reacts more to the last part of Turn 3 and defines the situation itself as positively something new. Maybe the client’s modesty triggers the professional to make another extended positive assessment: “*And as you apparently master well this jewelry making, after all, it is great, then you get to share your own expertise with others.*” This constructs the client as a skillful and valuable community member, who has know-how to be shared with others. Interestingly, the client once again ignores the positive feedback and compliment and moves to reflect on the good timing of her current project (organizing things around the home). One element of RIM includes advancing client’s belief in his/her capacities and value as a beneficial member of the community. Furthermore, positive assessments like those provided in this example may be interpreted as reinforcing the cultural expectation that human well-being is related to integration and having valuable roles in the community [8].

## 4. Discussion

### 4.1. Summary of the Results

The study identified the role of positive assessments in defining and promoting RIM at the grassroots level of mental health work, particularly during home visits. The way in which positive assessment is constructed affects the flow of interaction and the client’s agency as an interlocutor. In the data the sequences of positive assessments were initiated in two different ways: either as client-initiated “good news” or as client-provided “good news” that is in response to the professional’s questions or advice. Minimal positive assessments were brief feedback from professionals included statements that indicated that the clients were on the “right track” The extended positive assessments were more profound and reflected the client’s overall progress and successes in life. The positive assessments also had a function of supporting a client’s self-determination and self-governing agency.

The wide spectrum of the elements of RIM becomes evident when looking closely at the content of the professionals’ positive assessments. From the data analyzed in this study, RIM can be divided into two upper-categories. The first upper-category “Encouraging Doing the Right Things” entailed discussion of ordinary and routine things in one’s everyday life. These are often culturally understood as part of a decent, healthy, and normal life at home and in the community outside of institutional care. In the data normal life included taking care of everyday matters, the home, and oneself. In addition, living in the communities was related to an expectation and requirement of “normal” behavior in various human encounters. The second upper-category “Encouraging the Right Kind of Personal Growth” reflected more demanding and profound personal skills and strengths from the clients (see [18]). The professionals supported the clients’ self-governing agency by encouraging them to perform the “right” kind of illness-management and to be a knowledgeable and skillful member in their community. These elements emphasize the idea of personal growth embedded in the RIM.

The categorization of the elements of RIM conducted in this study is consistent with various classifications presented in previous recovery research [13,14,15]. RIM is challenging for both the clients and the floating support professionals because it requires transformation in both: when conducting the clients’ everyday routines at home and in the way the clients see themselves as members of the community. RIM covers all aspects of the clients’ lives, and makes these aspects the shared interest and responsibility of both the clients and professionals.

### 4.2. Culturally Laden, Negotiable, and Interactional RIM

The analysis suggests that positive assessments are used for strengthening the normal and preferred “things to do” or “agents to be” in Western culture. They are used for identifying and expressing the boundaries between normality and abnormality. Thus, positive assessments guide a person in a preferred direction in life. This finding is complementary to previous research, which has described how positive assessments are used to express empathy, understanding, or encouragement in order to restore a person’s coping (e.g., [27,28,30]).

This critical, cultural aspect of the analysis adds to the previous research of the elements of recovery (e.g., [3,9,12,13,14,15,16]) as it reveals the interpretative essence of RIM. It raises critical questions. Does the “recovery in” process emphasize more personal than societal transformation? Would more flexible cultural norms promote RIM better than educating and counseling the disadvantaged citizens to adjust better to existing society and its requirement? What is sufficient progress in “recovery in” processes?

The results also highlight how putting RIM into practice is not only about reflecting cultural but also situational and individual expectations. At each home visit, the professional and client discuss what it means for individual client to be in the recovery process. RIM is thus a locally negotiated and personalized concept. From this point of view, RIM has the potential to challenge cultural expectations and to produce alternative avenues to recovery. This potential is evident in our examples: for instance, in the sequence where the professional encourages the client not to participate in the activity that he does not prefer, even though participation would be in line with the aim of active citizenship and integration.

### 4.3. Critical Remarks and Limitations of the Study

This research design, as all research designs, has both strengths and limitations. For example, the study does not capture the participants’ inner emotions, sentiments, or experiences of RIM but only elements of the situational meaning making in professional-client interaction. The elements of RIM presented are a result of a qualitative analysis process with quite a small data corpus. When using a small data corpus and illustrative data examples, it is not possible to make extensive generalizations. We cannot be confident of the transferability of the results to the population as a whole. From the analysis it is not possible to make conclusions about the long-term influences of supportive home visit communication that includes positive assessments. For example, a qualitative longitudinal study could instead provide more information about how “in situ” assessments enhance the elements of RIM over time and whether RIM makes a difference in the clients’ lives. This study indicates that professional communication is meaningful “in situ” because the clients engage with the professionals in constructing the elements of RIM regarding their own lives.

## 5. Conclusions and Practical Implications

The study indicates that RIM is related to human interaction in the mental health recovery setting in multiple profound ways. RIM is deeply embedded in cultural meaning-making and in interpersonal interaction. Hence, RIM can be further developed to be a more socially, interactively, and culturally sensitive concept. RIM not only promotes the clients’ adjustment to current society but also questions the current expectations of active citizenship in Western society. The study contributes to the ongoing discussions on the elements of RIM.

This study has demonstrated how skillfully professionals use positive assessments in supportive home visit interactions, although they do not always do so consciously. To conclude, the categorization of the elements of RIM could be used in educating practitioners to identify and operationalize RIM in mental health home visits. The results can also be utilized to raise professionals’ awareness about interactional skills and positive assessments as working tools. Analyzing grass-roots level professional-client interactions makes the content of professional mental health work more visible and transparent for mental-health policymakers and managers.

## Figures and Tables

**Table 1:** Elements of “recovery in” model.

Upper-Category	Sub-Categories	Elements of ’Recovery in’ Model in Sub-Categories
Encouraging Doing the Right Things	Taking care of everyday matters	Financial issues and commitment to agreed appointments.
	Taking care of home	Cleaning: washing dishes, vacuuming, laundry, taking trash out.
	Taking care of oneself	Personal hygiene, medication, exercising, healthy eating, sufficient sleeping.
	Behaving in a normal way in the community	Appropriate behavior and social participation.
Encouraging Right Kind of Agency	Doing life planning	Continuality, contemplating and committing to future plans.
	Doing illness management	Recognising symptoms and own ways to manage with the illness.
	Being self-governing and knowing agent	Recognising own strengths and will.
	Being skillful community member	Recognising own skills and wishes to participate in communal actions.
